# Does labour market disadvantage help to explain why childhood circumstances are related to quality of life at older ages? Results from SHARE

**DOI:** 10.1080/13607863.2014.938604

**Published:** 2014-07-17

**Authors:** Morten Wahrendorf, David Blane

**Affiliations:** ^a^ Centre for Health and Society, Institute for Medical Sociology, University of Düsseldorf, Düsseldorf, Germany; ^b^ Department of Epidemiology and Public Health, International Centre for Life Course Studies in Society and Health, University College London, London, United Kingdom

**Keywords:** CASP, life course, labour market disadvantage

## Abstract

There is robust evidence that childhood circumstances are related to quality of life in older ages, but the role of possible intermediate factors is less explored. In this paper, we examine to what extent associations between deprived childhood circumstances and quality of life at older ages are due to experienced labour market disadvantage during adulthood. Analyses are based on the Survey of Health Ageing and Retirement in Europe (SHARE), with detailed retrospective information on individual life courses collected among 10,272 retired men and women in 13 European countries (2008–2009). Our assumption is that those who have spent their childhood in deprived circumstances may also have had more labour market disadvantage with negative consequences for quality of life beyond working life. Results demonstrate that advantaged circumstances during childhood are associated with lower levels of labour market disadvantage and higher quality of life in older ages. Furthermore, results of multivariate analyses support the idea that part of the association between childhood circumstances and later quality of life is explained by labour market disadvantage during adulthood.

## Introduction

Today's older people will live longer than any previous generation in Europe. In most European countries a 60-year-old woman or man can expect to live another 20 years, and in some of these even longer (Eurostat, 2013). This development is combined with the hope that the prolonged length of life is accompanied by good subjective quality of life. Unfortunately, for many older women and men, longer lives do not lead to this positive scenario, but rather to prolonged periods of morbidity (Wahrendorf, Reinhardt, & Siegrist, 2013) and lower levels of quality of life (Niedzwiedz, Katikireddi, Pell, & Mitchell, 2014; von dem Knesebeck, Wahrendorf, Hyde, & Siegrist, 2007). It has been shown that this is particularly the case for those who experienced less advantaged social and economic circumstances at earlier stages of their life course (Berney et al., 2000; Brandt, Deindl, & Hank, 2012; Mc Munn, Breeze, Goodman, & Nazroo, 2006; von dem Knesebeck et al., 2007 BreezeGoodmanNazroo), including adulthood and childhood social position (for a review see Niedzwiedz, Katikireddi, Pell, & Mitchell, 2012). However, few life course studies address possible pathways and ask what intermediate factors may explain the association between early disadvantage and later quality of life. For instance, those who have spent their childhood in deprived circumstances may also have had particular employment histories and thereby been exposed to more labour market disadvantage over the life course, with long-term consequences for quality of life.

In fact, studies have documented that social position in early life exerts important effects on educational achievements (Duncan, Yeung, Brooks-Gunn, & Smith, 1998), as well as on employment histories and the risk of labour market disadvantage later on. More specifically, research found effects of childhood poverty on youth unemployment (Caspi, Wright, Moffitt, & Silva, 1998), financial difficulties in early and middle adulthood (Kuh, Head, Hardy, & Wadsworth, 1997; Kuh & Wadsworth, 1991), job insecurity (Power & Matthews, 1997) and higher level of psychosocial stress at work (Elovainio et al., 2007). These findings are in line with existing ideas of cumulative disadvantages over the life course, where early disadvantage leads to an accumulation of subsequent disadvantages (Dannefer, 2003; Ferraro & Shippee, 2009). At the same time, there is increasing evidence that work and employment-circumstances over the life course are related to health and quality of life beyond working life, in particular for men. This includes unstable working careers, periods of unemployment and poor psychosocial conditions (Schröder, 2011b; Wahrendorf et al., 2013), as well as physical hazards at work (Platts et al., 2013).

Yet, as most studies are based on prospective cohorts (particularly birth cohorts that have yet to reach old age) the complex interrelations between childhood circumstances, labour market disadvantages and quality of life beyond working life are still relatively unexplored. More specifically, information about employment histories is restricted to either early or middle adulthood (Blane, Wahrendorf, Webb, & Netuveli, 2012), without information on quality of life beyond work (in the case of birth cohorts), or work-related factors are limited to the recent past without information on childhood circumstances (in the case of occupational cohorts). This leaves a gap of knowledge about longer term effects of adversity in early life on people's occupational careers and, additionally, about their effects on quality of life after labour market exit. An attempt to overcome this limitation is to use retrospective data, asking older men and women who already left the labour market about previous childhood conditions and entire employment history. In fact, there has been important methodological progress in collecting such data (Blane, 1996), and current research has shown promising findings (Börsch-Supan, Brandt, Hank, & Schröder, 2011; Börsch-Supan, Brandt, & Schroder, 2013). For example, one recent study used retrospective information on employment histories to show that education (usually related to social position in early midlife) is associated with higher levels of labour market disadvantage throughout working life, in terms of involuntary job loss, unemployment and a disadvantaged occupational position (Dragano & Wahrendorf, 2014). In this paper, we set out to extend this research by additionally including indicators of early childhood circumstances (Chittleborough, Baum, Taylor, & Hiller, 2006; Galobardes, Lynch, & Davey Smith, 2004), and by studying their links with quality of life after labour market exit.

### Aims

Along these lines, the first aim of this article is to study the association between deprived childhood circumstances and quality of life in older ages. Quality of life is measured by a short version of the CASP questionnaire (Hyde, Wiggins, Higgs, & Blane, 2003). Drawing on the literature of ageing (Laslett, 1996) and a theory of human needs (Doyal & Gough, 1991), this measure defines quality of life as the degree to which four human needs are satisfied: control, autonomy, self-realization and pleasure (see ‘Methods’ section for conceptual details). As a second aim, we study to what extent an association between childhood circumstances and quality of life can be explained by labour market disadvantage over working life. We hypothesize that children growing up in a context of socioeconomic adversity are more likely to face labour market disadvantage over working life and that these conditions enhance the probability of lower quality of life after labour market exit. This assumption follows the existing framework of cumulative disadvantages over the life course, where early advantages or disadvantages shape individual trajectories over the life course and lead to an accumulation of risk factors over the life course, with long-lasting effects on quality of life in later life (Dannefer, 2003; Ferraro & Shippee, 2009).

Taken together we study the two interrelated research questions:

1. Is there an association between childhood circumstances and quality of life at older ages?
2. If so, to what extent can this association be explained by labour market disadvantage during adulthood?

## Methods

### Data sources

We used third wave data from the Survey of Health, Ageing and Retirement in Europe (SHARE), collected during 2008–2009, that we combined with information on quality of life assessed in wave 2 from 2006 to 2007. SHARE is the first cross-national research project collecting data on a variety of sociological, economic and health-related topics among older adults in Europe. The survey started in 2004–2005 in 11 countries (Sweden, Denmark, Germany, the Netherlands, Belgium, France, Switzerland, Austria, Italy, Spain and Greece), with ongoing waves of data collection in two-year intervals. Two new countries joined SHARE in wave 2 (Czech Republic and Poland). In each country, samples consist of a probability household sample, with individuals aged 50 years or older plus their (possibly younger) partners. New cohorts (so-called ‘refreshers’) are added subsequently to maintain population representation. In contrast to waves 1 and 2, the third wave of SHARE consists in a detailed retrospective assessment of respondents’ previous life (also called SHARELIFE) (Börsch-Supan et al., 2013). This includes information on childhood and previous employment histories among those who have left the labour market. With regard to survey participation, response rates of SHARE are generally above average compared to other European surveys (Börsch-Supan & Jürges, 2005). At study onset the household response rates were 61% for the total sample ranging from 81% in France to 39% in Switzerland, with rates above 50% in eight countries. With respect to attrition between waves 2 and 3, the percentage of respondents lost varied between 34% (Austria) and 14% (Switzerland), with rates below 20% in seven countries (Schröder, 2011a). Retrospective data were collected by a lifegrid, where recall and timing of major information is supported by a graphical representation of a respondent's life, filled in during the course of the interview. The method was developed first as a self-completion questionnaire (Blane, 1996), and subsequently transformed into a Computer Assisted Personal Interviews (CAPI) by the UK National Centre for Social Research (Scholes et al., 2009). The latter was adopted for SHARELIFE (Schröder, 2011a). Although recall bias is a disadvantage of data based on retrospective questions, this approach has several advantages. First, it represents a fast and less expensive method to obtain longitudinal information. Second, it guarantees comparable information referring to different time points in respondents’ life histories (without missing data due to panel attrition). Third, validation studies revealed high accuracy of recalled information, in particular when asking about socio-demographic conditions (Berney & Blane, 1997; Havari & Mazzona, 2011) and employment histories (Baumgarten, Siemiatycki, & Gibbs, 1983; Bourbonnais, Meyer, & Theriault, 1988). More details about SHARE and its methods are available online (www.share-project.org).

### Respondents

In total, 26.836 participants were interviewed at wave 3. For the analyses we considered only people who had left the labour market when measuring quality of life. This serves our aim to study quality of life beyond working life. Furthermore, respondents were only included if they documented an employment history of at least five years. Otherwise, information on previous employment histories was not considered to be of sufficient importance. Moreover, we excluded respondents older than 80 years when answering the lifegrid questionnaire. This restriction helped to avoid a sample bias because people over 80 years may have had more favourable employment histories with later mortality (all analyses were calculated with a sample including people over 80 years as well, but findings remain unchanged). Finally, we excluded respondents when the interviewer documented respondent difficulties in answering the lifegrid questionnaire (about 4% of the total sample). These restrictions resulted in a final sample with full available data of 4808 men and 5463 women (*N* = 10,271) born between 1928 and 1947.

### Measures

#### Quality of life in older ages

Quality of life was measured by CASP-12v.1, a short version of the CASP-19 questionnaire. One of the innovations of SHARE was the inclusion of this measure – a psychometrically validated short version of the original 19 item version (CASP-19) (Hyde et al., 2003; Wiggins, Netuveli, Hyde, Higgs, & Blane, 2008). An important characteristic of this instrument is that it does not focus on respondents’ self-evaluation of quality of life, nor does it measure quality of life using measures of health as proxies. It rather identifies four domains of human needs (Doyal & Gough, 1991) that are relevant in later life. These needs refer to different strands of the literature on ageing; first, the new opportunities of the third age compared to former stages in life (Laslett, 1996), and second, the literature of Giddens (1991) and the role of older people in a rapidly changing society. The four domains are: control (C), autonomy (A), self-realization (S) and pleasure (P). The experience of these aspects (over the past four weeks) is measured with 12 questionnaire items (three for each domain) which are scored on a four-point Likert scale. A summary measure of the 12 items is used to assess quality of life in this study where the total sum score ranges from 0 to 36, with higher scores indicating better quality of life. In our sample Cronbach's alpha was 0.81 for men and 0.82 for women. Details on psychometric properties of CASP-19 and on its conceptual basis are fully described elsewhere (Higgs, Hyde, Wiggins, & Blane, 2003; Hyde et al., 2003).

#### Childhood circumstances

This variable is measured by an index combining four binary indicators of adverse socio-economic conditions during childhood. All single measures reflect the respondents’ conditions when they were 10 years old. The following items were used, all based on measures of previous studies that assessed the long-term effects of childhood social position on health during adulthood (Chittleborough et al., 2006; Dedman, Gunnell, Smith, & Frankel, 2001; Evans, Kelley, Sikora, & Treiman, 2010; Marsh, 1999). First, we included the occupational position of the main breadwinner, as assessed by the 10 main occupational groups of the International Standard Classification of Occupations (ISCO). As in a previous article (Wahrendorf, Blane, Bartley, Dragano, & Siegrist, 2013), these groups were reclassified according to the different skill levels, representing the broad hierarchical structure of ISCO, which we regrouped into low (first and second skill levels) and high (third and fourth skill levels) occupational positions. Second, respondents were asked to report the number of books at home, using the category ‘less than 10 books’ as an indicator of social disadvantage (Evans et al., 2010). Third, a measure of overcrowding was generated by combining information on the number of people living in the household with number of available rooms (excluding kitchen, bathrooms and hallways). Overcrowding was coded in all cases where more than one person per room lived in the household (Marsh, 1999). Finally, housing quality was explored, where poor quality was rated when none of the following characteristics was available: fixed bath, cold running water supply, hot running water supply, inside toilet and central heating (Dedman et al., 2001). Based on this information, a five-categorical variable of childhood circumstances was constructed, ranging from ‘most advantaged’ to ‘most disadvantaged’.

#### Labour market disadvantage

From detailed information on individual employment histories available in SHARELIFE we developed an index of labour market disadvantage, based on the following four items. The first item asked whether an involuntary job loss occurred as a consequence of being laid off. With the second item, involuntary job loss due to plant closure was assessed. Third, we measured the occupational position in respondents’ main job, again based on the ISCO classification (which we regrouped into two categories ‘low and high occupational position’ as described above). With the fourth item an episode of unemployment lasting at least six months was registered. By combining these four items, we defined five possible levels of labour market disadvantage, ranging from ‘none’, ‘mild’, ‘moderate’, ‘severe’ to ‘very severe’ disadvantage.

#### Additional variables

In addition to age and sex, we included functional limitations, education and a variable measuring respondents’ partnership history. Functional limitation was measured in the same year when measuring quality of life with the Global Activity Limitation Indicator (GALI) index (Jagger et al., 2010). Education was measured according to the International Standard Classification of Educational Degrees (ISCED-97) that we regrouped into ‘low education’ (pre-primary, primary or lower secondary education), ‘medium education’ (secondary or post-secondary education) and ‘high education’ (first and second stage of tertiary education). In the case of partnership history, we combined information of whether the respondents lived with a partner at the age of 30 and 50 (without considering the marital status), resulting in a four-categorical variable.

### Analyses

**Figure 1.**
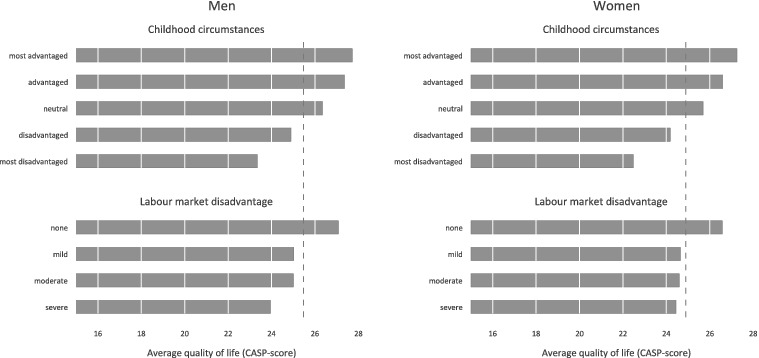
Quality of life by childhood circumstances and labour market disadvantage for men (*N* = 4808) and women (*N* = 5463). Note: Dashed line presents overall averages in quality of life for men and women.

**Figure 2.**
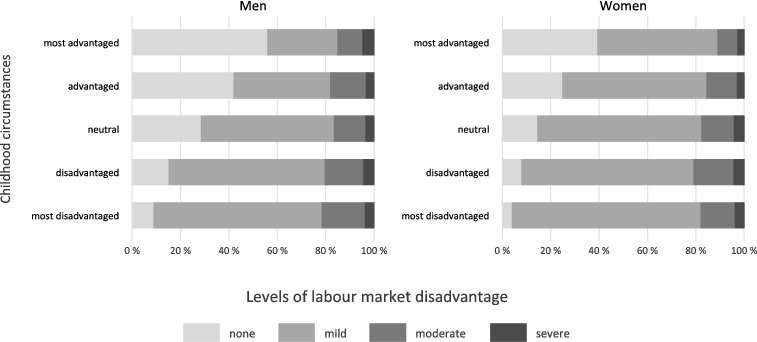
Percentages of labour market disadvantage by childhood circumstances for men (*N* = 4808) and women (*N* = 5463).

All analyses are conducted for men and women separately and we start with a basic sample description (Table 1). Then we present average scores of quality of life (mean CASP score) by childhood circumstances and levels of labour market disadvantage (Figure 1). In these (and subsequent) analyses the two highest levels of labour market disadvantage (‘severe’ and ‘very severe’) were combined due to low frequencies, thus, leading to four categories. In the following, we study if deprived childhood circumstances are related to more disadvantaged labour market histories (Figure 2).

**Table 1.** Sample description: percentages and frequencies (*N*) or mean scores and standard deviation (SD) for men and women (*N* = 10,271).

		Men *N* = 4808		Women *N* = 5463	
Variables	Categories or range	% or (mean)	*N* or (SD)	% or (mean)	*N* or (SD)
CASP	0–36	25.5	(6.1)	24.9	(6.3)
					
Age	50–80	68.9	(6.3)	66.9	(7.1)
					
Partner	At 30 and 50	79.3	3812	83.8	4580
	At 30 but not 50	2.7	131	5	273
	Not at 30 but 50	12.9	620	5.9	323
	Not at 30 and 50	5.1	245	5.3	287
					
Functional limitations	Not limited	57.5	2766	52.5	2876
	Limited	42.5	2042	47.5	2596
					
Education	Low	46.8	2251	53.0	2898
	Medium	34.1	1638	33.4	1823
	High	19.1	919	13.6	742
					
Childhood circumstances	Most advantaged	4.8	231	4.5	247
	Advantaged	15.1	724	16.5	902
	Neutral	29.4	1415	33.7	1844
	Disadvantaged	27.3	1312	25.7	1402
	Most disadvantaged	23.4	1126	19.5	1068
					
Labour market disadvantage	None	23.6	1134	13.6	741
	Mild	57.5	2763	68.5	3742
	Moderate	15.1	726	14.0	766
	Severe	3.3	161	3.5	192
	Very severe	0.5	24	0.4	22

We then estimate a series of multilevel linear models using quality of life as dependent variable with individuals (level 1) nested in countries (level 2) (Rabe-Hesketh & Skrondal, 2005). Using multilevel modelling allows for accurate adjustment for country affiliation, because the constant is allowed to vary across countries. This is important for our analyses, because of previously reported country variations of quality of life in SHARE (von dem Knesebeck et al., 2007). In addition, variations of quality of life can be studied at each level separately (within- and between-country variations). In sum, we estimate five different models. The first model contains a constant term only and quantifies the amount of variation of quality of life at each level (empty model). Models 1 and 2 present the adjusted effects for childhood circumstances (Model 1) and levels of labour market disadvantage (Model 2), both included as categorical variables (broken into dummies) and adjusted for sex, age, age square and partnership history. In Model 3 we additionally include labour market disadvantage and study our main research questions, that is, to what degree the association between deprived childhood circumstances and quality of life is explained by labour market disadvantage. In addition, we perform a formal test of mediation for multilevel models (Krull & MacKinnon, 2001) and test the significance of an indirect effect of childhood disadvantages via labour market disadvantage (both treated linear in this case) based on bootstrapping with 5000 replications. In Model 4 we finally include functional limitations and education. Results of multilevel regressions are presented in Table 2 for women and Table 3 for men, where we present the estimated unstandardized regression coefficients, together with standard errors and level of statistical significance. For each model the log likelihood, the AIC (Akaike Information Criterion) and the BIC (Bayesian Information Criterion) statistics are indicated, and the proportional reduction of variance explained at each level (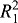, 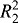) is reported (Snijders & Bosker, 1994).

**Table 2.** Multilevel estimates for quality of life in older ages for women: regression coefficients (*b*) and standard errors (SE) (*N* = 5463).

				Model 1		Model 2		Model 3		Model 4	
Model		Empty model		*b*	(SE)	*b*	(SE)	*b*	(SE)	*b*	(SE)
Fixed parameters											
Age				1.21***	(0.19)	1.09***	(0.18)	1.14***	(0.19)	0.76***	(0.18)
Age square				−0.01***	(0.00)	−0.01***	(0.00)	−0.01***	(0.00)	−0.01***	(0.00)
Partner	At 30 and 50			–		–		–		–	
	At 30 but not 50			−2.14***	(0.35)	−2.08***	(0.35)	−2.16***	(0.35)	−2.03***	(0.33)
	Not at 30 but 50			−0.95**	(0.32)	−0.84**	(0.32)	−0.96**	(0.32)	−0.89**	(0.30)
	Not at 30 and 50			−1.28***	(0.34)	−1.17***	(0.34)	−1.29***	(0.34)	−1.19***	(0.32)
Childhood circumstances	Most advantaged (ref.)			–				–		–	
	Advantaged			−0.29	(0.40)			−0.11	(0.40)	−0.05	(0.38)
	Neutral			−0.60	(0.38)			−0.27	(0.39)	−0.10	(0.37)
	Disadvantaged			−1.36***	(0.39)			−0.94*	(0.40)	−0.35	(0.38)
	Most disadvantaged			−2.08***	(0.42)			−1.60***	(0.42)	−0.81*	(0.41)
Labour market disadvantage	None (ref.)					–		–		–	
	Mild					−1.52***	(0.23)	−1.23***	(0.23)	−0.54*	(0.24)
	Moderate					−1.67***	(0.29)	−1.37***	(0.30)	−0.72*	(0.30)
	Severe					−2.46***	(0.44)	−2.12***	(0.44)	−1.26**	(0.43)
Functional limitations	Not limited									–	
	Limited									−3.84***	(0.15)
Education	Low									–	
	Medium									0.69***	(0.18)
	High									1.16***	(0.27)
											
Random parameters											
Level 1: within country		5.69***	(0.05)	5.60***	(0.05)	5.60***	(0.05)	5.58***	(0.05)	5.24***	(0.05)
Level 2: between country		2.67***	(0.53)	2.51***	(0.50)	2.73***	(0.54)	2.54***	(0.50)	2.53***	(0.50)
											
Statistics											
(level 1)				.033		.033		.039		.152	
(level 2)				.120		−.042		.101		.105	
Log likelihood		−17,284.75		−17,192.64		−17,194.52		−17,174.42		−16,830.68	
AIC		34,575.50		34,409.28		34,411.04		34,378.84		33,697.37	
BIC		34,595.32		34,488.55		34,483.71		34,477.93		33,816.27	

Note: The ICC of the empty model is 0.18. *p < 0.05; **p < 0.01; ***p < 0.001.

**Table 3.** Multilevel estimates for quality of life in older ages for men: regression coefficients (*b*) and standard errors (SE) (*N* = 4808).

				Model 1		Model 2		Model 3		Model 4	
Model		Empty Model		*b*	(SE)	*b*	(SE)	*b*	(SE)	*b*	(SE)
Fixed parameters											
Age				1.49***	(0.24)	1.28***	(0.24)	1.33***	(0.24)	0.87***	(0.22)
Age square				−0.01***	(0.00)	−0.01***	(0.00)	−0.01***	(0.00)	−0.01***	(0.00)
Partner	At 30 and 50			–		–		–		–	
	At 30 but not 50			−0.73	(0.49)	−0.53	(0.49)	−0.61	(0.49)	−0.57	(0.46)
	Not at 30 but 50			−0.14	(0.24)	−0.11	(0.24)	−0.14	(0.24)	−0.07	(0.23)
	Not at 30 and 50			−0.99**	(0.36)	−0.87*	(0.36)	−0.90*	(0.36)	−0.89**	(0.34)
Childhood circumstances	Most advantaged (ref.)			–				–		–	
	Advantaged			−0.21	(0.42)			−0.12	(0.41)	−0.10	(0.39)
	Neutral			−0.57	(0.39)			−0.37	(0.39)	−0.29	(0.37)
	Disadvantaged			−1.28**	(0.40)			−0.97*	(0.40)	−0.48	(0.39)
	Most disadvantaged			−1.89***	(0.42)			−1.52***	(0.42)	−0.89*	(0.41)
Labour market disadvantage	None (ref.)					–		–		–	
	Mild					−1.04***	(0.20)	−0.77***	(0.21)	−0.22	(0.21)
	Moderate					−1.32***	(0.27)	−1.04***	(0.27)	−0.58*	(0.26)
	Severe					−2.59***	(0.44)	−2.30***	(0.45)	−1.63***	(0.43)
Functional limitations	Not limited									–	
	Limited									−3.76***	(0.15)
Education	Low										
	Medium									1.07***	(0.19)
	High									1.08***	(0.25)
											
Random parameters											
Level 1: within country		5.55***	(0.06)	5.49***	(0.06)	5.49***	(0.06)	5.47***	(0.06)	5.14***	(0.05)
Level 2: between country		2.42***	(0.48)	2.16***	(0.43)	2.34***	(0.47)	2.16***	(0.43)	2.10***	(0.42)
											
Statistics											
(level 1)				.020		.021		.027		.141	
(level 2)				.201		.062		.201		.247	
Log likelihood		−15,085.98		−15,036.44		−15,035.61		−15,019.49		−14,719.39	
AIC		30,177.97		30,096.88		30,093.22		30,068.99		29,474.78	
BIC		30,197.40		30,174.62		30,164.48		30,166.16		29,591.38	

Note: The ICC of the empty model is 0.16. *p < 0.05; **p < 0.01; ***p < 0.001.

**Figure 3.**
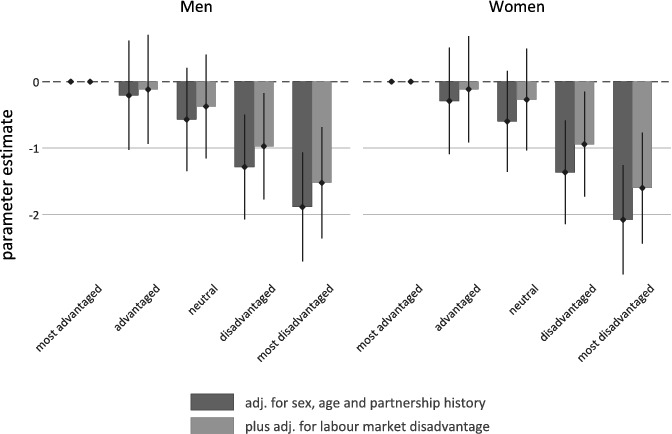
Childhood circumstances and quality of life in older ages: multilevel estimates and confidence intervals (95%) for men (*N* = 4808) and women (*N* = 5463). Note: Estimates are based on Models 1 and 3 from Tables 2 and 3.

In a final step, we summarize the main findings of our study for both men and women in Figure 3, where estimates of childhood circumstances are presented – before and after adjustments for labour market disadvantage.

## Results

### Sample description

Our sample included slightly less men than women (4808 men vs. 5463 women). Men were on average two years older (69 vs. 67 years) at the time of the SHARELIFE interview. Quality of life was slightly better for men as compared to women. No systematic differences between men and women were found for the remaining variables, except for men reporting slightly higher levels of education and being more likely to report functional limitations (see Table 1 for details). Levels of labour market disadvantage were rather low, with less than 1% experiencing very severe disadvantage only. Therefore, subsequent analyses combined the two highest levels of labour market disadvantage.

### Quality of life by childhood circumstances and labour market disadvantage

Figure 1 displays average scores of quality of life by childhood circumstances and labour market disadvantage. In the case of childhood circumstances, for both men and women we observe a clear graded association, with social disadvantage related to lower quality of life at older ages. Similarly, the more men or women experienced labour market disadvantage during working life, the lower is their quality of life after labour market exit.

### Childhood circumstances and labour market disadvantage

Are deprived childhood circumstances related to more labour market disadvantage during working life? An answer to this question is given in Figure 2. We see that the labour market disadvantage ‘none’ is more frequent among men and women with advantaged childhoods. More specifically, more than half the men with the most advantaged childhoods reported no labour market disadvantage (about 40% in the case of women).

### Results of multivariate analyses

Results of multilevel analyses are presented for women in Table 2 and for men in Table 3. In both cases, the empty model shows significant variations of the standard deviations at individual and at country level, with an intra-class correlation (ICC) of 0.16 for men and 0.18 for women. This indicates that most of the variations in quality of life are due to differences between individuals rather than countries in our sample.

Turning to the fixed parameter of the two tables, four observations deserve attention. First, for both men and women we see a stepwise decrease of the regression coefficients of childhood circumstances (Model 1) and of labour market disadvantage (Model 2), where deprived circumstances during childhood (or higher levels of labour market disadvantage) are related to lower quality of life after retirement. This confirms findings of Figure 1. Second, we observe that effects of partnership histories on quality of life differ between men and women. Although the quality of life was the best for both men and women if they lived with a partner at the age of 30 and 50 years (reference category), the effect of living without a partner was stronger in the case of women. The third observation worth noting refers to our core research question and Model 3, where childhood circumstances and labour disadvantage are combined into one model. The regression coefficients of childhood circumstances are generally attenuated, but remain significant for the two most deprived categories (again for men and women). According to tests of mediation (not shown in the tables), the indirect effects are significant for both men (*z* = −5.26, *p* < 0.001) and women (*z* = −4.69, *p* < 0.001). This indicates that part of the association between deprived childhood circumstances and quality of life is explained by labour market disadvantage. Finally, when including functional limitation and education in Model 4, coefficients for childhood circumstances are again attenuated and, additionally, the coefficients for labour market disadvantage are reduced. On the one hand, this suggests that functional limitations and educational qualification may be additional intermediate factors linking childhood adversity and quality of life for both men and women. On the other hand, in the case of education, it is also thinkable that it acts as a confounder, where education (because of its stability over the life course) affects both labour market disadvantage and quality of life in older ages.

With respect to the random parameters, model fits are the best in the final models. In the case of women, the *R* ^2^ statistics suggest that the considered variables explain about 15% of the variations at the individual level (14% for men) and 10% between country variations (25% for men).

To summarize our main results, Figure 3 presents a visual summary of the estimated coefficients for childhood circumstances together with confidence intervals – before and after adjustments for labour market disadvantage.

## Discussion

In this paper, we studied the association between childhood circumstances and quality of life after labour market exit. In addition, we used detailed information on previous employment histories, and studied the extent to which labour market disadvantage can explain an association. Main findings can be summarized as follows.

With regard to our first research question, we found strong support that deprived childhood circumstances are related to lower quality of life after labour market exit, with a clear gradient for both men and women: the more disadvantaged people's circumstances during childhood, the more likely they are to report lower quality of life in older ages. Similarly, those who grew up in deprived circumstances were more likely to experience higher levels of labour market disadvantage. These findings are in line with previous research (Holland et al., 2000; Kuh et al., 1997; Kuh & Wadsworth, 1991; Niedzwiedz et al., 2012; Power & Matthews, 1997), but two new elements may be added. First, by using different indicators to measure disadvantages during childhood, we were enabled to discover a cumulative impact of childhood deprivation on quality of life. Second, we used information on labour market disadvantage that covered entire employment history and, thus, extended the time frame to entire working careers.

With regard to the second question, associations between childhood social position and quality of life were weakened in multivariate models once labour market disadvantage was introduced. This weakened, yet statistically significant, effect points to a partial mediation, indicating that children who grew up in disadvantageous circumstances were more likely to experience labour market disadvantage; this partly explains their lower quality of life beyond working life. Again, as these analyses were based on detailed information of childhood social position and labour disadvantage throughout working life, this finding adds to existing literature. On a conceptual level, findings are in line with the existing framework of cumulative disadvantages over the life course and its origin during early childhood (Dannefer, 2003; Ferraro & Shippee, 2009).

We found two additional findings. First, we observed that effects of partnership histories on quality of life differ between men and women, where the negative impact of living without partnership during working life appeared more consistent in the case of women. This may indicate a higher importance of partnerships for women (at least in our sample). Second, although the focus of this paper was on labour market disadvantage, we found that educational qualification and functional limitations may be additional intermediate factors on the causal chain linking childhood circumstances and quality of life at older ages. Although these latter findings deserve more detailed analysis, this again supports the idea that childhood adversity leads to cumulative disadvantages during the life course.

When interpreting the results, we must consider the following limitations. First, the data measuring childhood circumstances and labour market disadvantage were assessed retrospectively. This fact carries the risk of systematic reporting bias. For example, information may be positively tuned due to a tendency of harmonizing conflicting retrospective biographical accounts. Yet, a high prevalence of disadvantaged childhood circumstances does not support this argument. Furthermore, the measure of labour market disadvantage was based on specific characteristics of the employment history (rather than self-perceived disadvantage). Finally, a recent study compared information collected in SHARELIFE and historical data at a national level and the results confirmed the validity of the retrospective data (Havari & Mazzona, 2011). Clearly, an additional inclusion of personality characteristics as confounders may have offered a more convincing case of tackling this limitation, but this information was not available in the data. Second, in this study we focussed on labour market disadvantage as one possible intermediate exposure at the structural level (Blane, Kelly-Irving, Errico, Bartley, & Montgomery, 2013) and, thus, we surely may have bypassed other important exposures during adulthood, including behavioural, material or psychosocial exposures (e.g. social support or work stress). Yet, we maintain that labour market disadvantage plays a crucial role, because many of these latter exposures are related to labour market disadvantage (e.g. higher levels of work stress or lower salary in the case of labour market disadvantage). However, future analyses are needed to disentangle these complex interrelations. Similarly, although our findings point to a cumulative impact of deprived childhood circumstances on quality of life, where each single indicator of deprived childhood circumstances is associated with lower quality of life (and with labour market disadvantage) (Dannefer, 2003), future analyses may test each single indicator separately as well. Furthermore, one may ask if our results can be generalized to other generations, because most men and women in our sample (born between 1908 and 1943) grew up under specific circumstances (e.g. 1930s – depression) and had specific employment histories (e.g. Second World War) (Elder, 1998). Therefore, the significance of our results needs to be evaluated in future studies for different generations. Similarly, although our multilevel models did consider country variations of quality of life, we may nevertheless ask if strengths of associations between social circumstances, labour market disadvantages and quality of life differ between countries. For example, one may assume that existing regulations and national policies may mitigate an effect, for example through policies offering social provision (decommodification), or – maybe even more importantly – through regulations of active labour market policies, as suggested in a recent study (Lunau, Wahrendorf, Dragano, & Siegrist, 2013). If further validated this latter aspect may point to possible policy implications of our findings. Finally, although our overall sample was relatively large, survey participation at study onset was not very high in some countries (e.g. Switzerland) and, thus, we cannot rule out that an unobserved selection bias affects our findings. For example, people with lower quality of life may be less likely to participate and, therefore, we may have overestimated levels of quality of life. Yet, studies showed that SHARE represents general populations quite well (Börsch-Supan & Mariuzzo, 2005), and it seems unlikely that participation rates may affect the reported associations in our study.

These limitations are balanced by important strengths. The SHARE study meets high-quality standards of data collection, specifically a vigorously controlled study protocol and comparable sample procedures in each country. Additionally, the survey uses validated questionnaires that have been translated into different languages following standard procedures (Schröder, 2011a). Finally, to our knowledge, this is the first survey that explicitly tests the complex interrelations between different indicators of childhood circumstances, labour market disadvantage and quality of life in older ages.

In conclusion, this study demonstrates that deprived childhood circumstances are related to lower quality of life in older ages and that this association is partly due to labour market disadvantage during working life. In other words, quality of life in older ages is related to childhood conditions. These conditions shape individuals’ life courses and their employment histories. These in turn carry the risk of lower quality of life beyond working life. Furthermore, the study illustrates the value of retrospective data in analysing determinants of quality of life in older age.
